# A Case of Aggressive Resuscitation and Timely Surgical Intervention to Reverse Severe Acidosis After Multiple Gunshot Wounds to the Chest, Abdomen, and Left Shoulder With a Bullet Fragment Retained in the Heart

**DOI:** 10.7759/cureus.16362

**Published:** 2021-07-13

**Authors:** Michael Rosenberger, Jonathan Lo, Gudata Hinika, Monika Shenouda, Moses Salibian

**Affiliations:** 1 Medical Education, Ross University School of Medicine, Miramar, USA; 2 Department of Surgery, California Hospital Medical Center, Los Angeles, USA

**Keywords:** massive hemorrhage, damage control resuscitation, damage control surgery, severe acidosis, gunshot, retained bullet, massive transfusion protocol, thoracotomy, laparotomy, lethal triad

## Abstract

The lethal triad of coagulopathy, hypothermia, and acidosis is a well-known cause of severe deterioration and poor prognosis in trauma patients. The presence of this triad complicates the surgical management of a patient suffering from penetrating injury and hemorrhage. Here, we report the case and management of a 44-year-old man with multiple high-caliber gunshot wound (GSW) injuries who became severely acidotic (pH <6.8) with hemorrhagic shock in the setting of massive hemorrhage due to penetrating chest and abdominal trauma. The patient sustained one high-caliber GSW to the left upper quadrant of the abdomen, one high-caliber GSW to the left periumbilical region of the abdomen, one high caliber GSW to the fourth intercostal space of the left chest just medial to the midclavicular line with an expanding hematoma, and one high-caliber GSW to the left shoulder with a floating left shoulder. He arrived at the Emergency Department conscious with a stable pulse but quickly became hemodynamically unstable. He required a thoracotomy and exploratory laparotomy in addition to a massive transfusion protocol. This case demonstrates the reversal of a severely acidotic patient due to massive hemorrhage to a blood pH within normal limits using damage control resuscitation surgery and massive transfusion protocols. The patient has since been discharged home in a stable condition with minimal long-term sequelae.

## Introduction

Massive transfusion protocols (MTPs) are initiated on a patient as a form of damage control resuscitation (DCR) to treat massive hemorrhage. Massive hemorrhage is defined as bleeding that requires a transfusion of >10 units of packed red blood cells (pRBCs) in 24 hours or >4 units of pRBCs in one hour, the replacement of the entire blood volume of the patient within 24 hours or greater than 50% of the entire blood volume within three hours, or a blood loss rate of greater than 150 mL/minute causing hemodynamic instability. Early clinical signs that can predict whether a patient might meet these criteria in an emergency setting include a heart rate of >120 beats per minute, systolic blood pressure <90 mmHg, positive focused assessment with sonography in trauma scan, and penetrating trauma. If one of the three massive hemorrhage criteria are met with the additional predictors indicated in the emergency setting, then DCR measures are indicated [1].

The goal of DCR is to achieve hemostasis while minimizing the loss of blood to gain a cardiac output that is compatible with survival. DCR strategies include early recognition and hemorrhage control during the transportation of the patient, damage control surgery to stop bleeding and minimize contamination, definitive surgery once hemostasis is initially achieved, and stabilization in an intensive care setting. MTPs are employed to replenish blood flow to the body while trauma surgeons manage surgical hemostasis [2-4].

MTP describes the ideal ratio of blood products used to replenish a patient who has massive hemorrhage and needs DCR. This ratio is defined as 1:1:1 (or 2:1:1) of pRBCs:platelets:plasma to optimize the patient’s blood with the goal of hemostasis. In addition to these products, supplemental calcium should be administered for every two units of pRBCs given. Additional products can be used as an adjunct to MTPs including tranexamic acid (TXA) and recombinant factor VIIa with frequent lab measurements to guide aggressive resuscitation measures. TXA is used to inhibit fibrinolysis and should be administered within three hours of the injury if needed, and recombinant factor VIIa enhances coagulation [5].

Another goal of MTP is to avoid the lethal triad of acidosis, hypothermia, and coagulopathy. It is imperative to prevent all three of these simultaneously to prevent the vicious cycle and subsequent deterioration of the patient’s condition. This can be achieved by warming fluids and blood products as they are transfused, warming the patient by increasing room temperature, applying external heating measures via a cloth or convective warming blankets to the limbs, and administering internal heating measures by running normal saline through surgical cavities in the abdomen or chest. In doing so, the heating measures prevent or reverse hypothermia and the transfusions prevent or reverse coagulopathy and acidosis [6,7].

## Case presentation

A 44-year-old Hispanic male with no known history was brought to the emergency department (ED) via Emergency Medical Services (EMS) after being shot in the chest, abdomen, and left shoulder. With EMS, the patient was conscious and had a stable pulse. In the trauma bay, the patient became unresponsive with a loss of pulse, and CPR was initiated. Upon physical examination, the patient was observed to have one high-caliber GSW to the left upper quadrant of the abdomen, one high-caliber GSW to the left periumbilical region of the abdomen, one high-caliber GSW to the fourth intercostal space of the left chest just medial to the midclavicular line with an expanding hematoma, and one high-caliber GSW to the left shoulder with a floating left shoulder. In the ED, a left thoracotomy, along with a central line and endotracheal intubation, was performed to release a pericardial tamponade that was likely caused by gunshot trauma to the heart. Cardiac massage and MTPs were initiated and an aortic cross-clamp was placed. Palpable pulses were achieved with the support of cardiac massage and MTPs after the release of a pericardial tamponade via pericardiotomy and multiple rounds of epinephrine. Following this ED course, the patient was immediately transported to the operating room (OR) for DCR surgery and MTP.

Upon arrival in the OR, the patient was aggressively resuscitated with high doses of epinephrine for vascular support. The thoracic cavity was surgically explored first. The patient was unstable due to active bleeding from a left hilar and left upper lobe injury. A left lung hilar clamp was placed to control the active bleeding while a tractotomy repair of the left lung injury was completed. The left hilar lung clamp was then released with minimal active bleeding from the site of the lung injury. Once this bleeding was controlled, an inspection of the remainder of the thoracic cavity began. At this time, the ascending arch and the descending portions of the aorta appeared intact. Upon further inspection, there was a hematoma and injury to the left subclavian vein which was immediately packed with gauze, and pressure was applied. Inspection and irrigation of the remainder of the thoracic cavity revealed no additional hemorrhagic injuries. During this time, the patient also received massive transfusions of pRBCs, platelets, and plasma.

Immediately after stabilizing the thoracic cavity of any hemorrhaging, exploration of the abdominal cavity commenced with a midline exploratory laparotomy incision. A liter of clotted blood was discovered upon opening the abdominal cavity. The four abdominal quadrants were initially inspected and significant injury to the omentum was found. Solid organs were inspected next, with no injuries observed to the liver, kidneys, spleen, or pancreas. Finally, the bowel was run from the distal esophagus to the colon with no evidence of injury along the alimentary tract. After careful inspection, the abdomen was packed to provide hemostasis for the omental injuries, and attention was focused back on the chest.

Significant oozing and bleeding from a tributary of the left subclavian vein were noted from the left shoulder upon reinspection of the chest. This tributary was clipped to control the active bleeding. Following this, the soft tissue bleeding surrounding the left chest and shoulder region was controlled via electrocautery and suture ligature. Further inspection of the left shoulder indicated an extensive left shoulder fracture with significant bone loss. Additionally, there was significant bleeding from the bone fracture itself. This was also packed with gauze for hemostasis, and a bullet fragment was removed from the left shoulder. After controlling the bleeding left chest and shoulder, a reinspection of the entire thoracic cavity indicated no further hemorrhage from the noted sites of injury or any additional sites.

After confirming that the patient was hemodynamically improving with high doses of epinephrine and continuous MTPs via arterial blood gas (ABG) and vital sign monitoring, the decision was made to attempt to release the aortic cross-clamp. To begin, the intrathoracic aortic cross-clamp was only partially released with good toleration from the patient. As the patient continued to tolerate this partial release, the sequential decision was made to release the intrathoracic aortic cross-clamp completely. At this time, the patient continued to hemodynamically tolerate the complete release of the cross-clamp; however, he was observed to have decreased chest movement of his right chest. A right thoracostomy tube was placed with 900 cc of bloody output. Attention was focused back on the abdomen for a reinspection that indicated no bleeding in addition to the ligated omental bleed. The abdominal cavity was then left open, packed with a wound vac sponge, and sealed with a wound vac in accordance with damage control surgery protocol. A reinspection of the thoracic cavity indicated no additional obvious bleeding, and hemodynamic values via vitals and ABG monitoring were stable at this point. The decision was made to close the thoracic cavity with the consent of the cardiothoracic surgeon who had arrived for a consultation. Two left chest tubes and one right chest tube were placed upon closure of the thoracic cavity.

**Figure 1:**
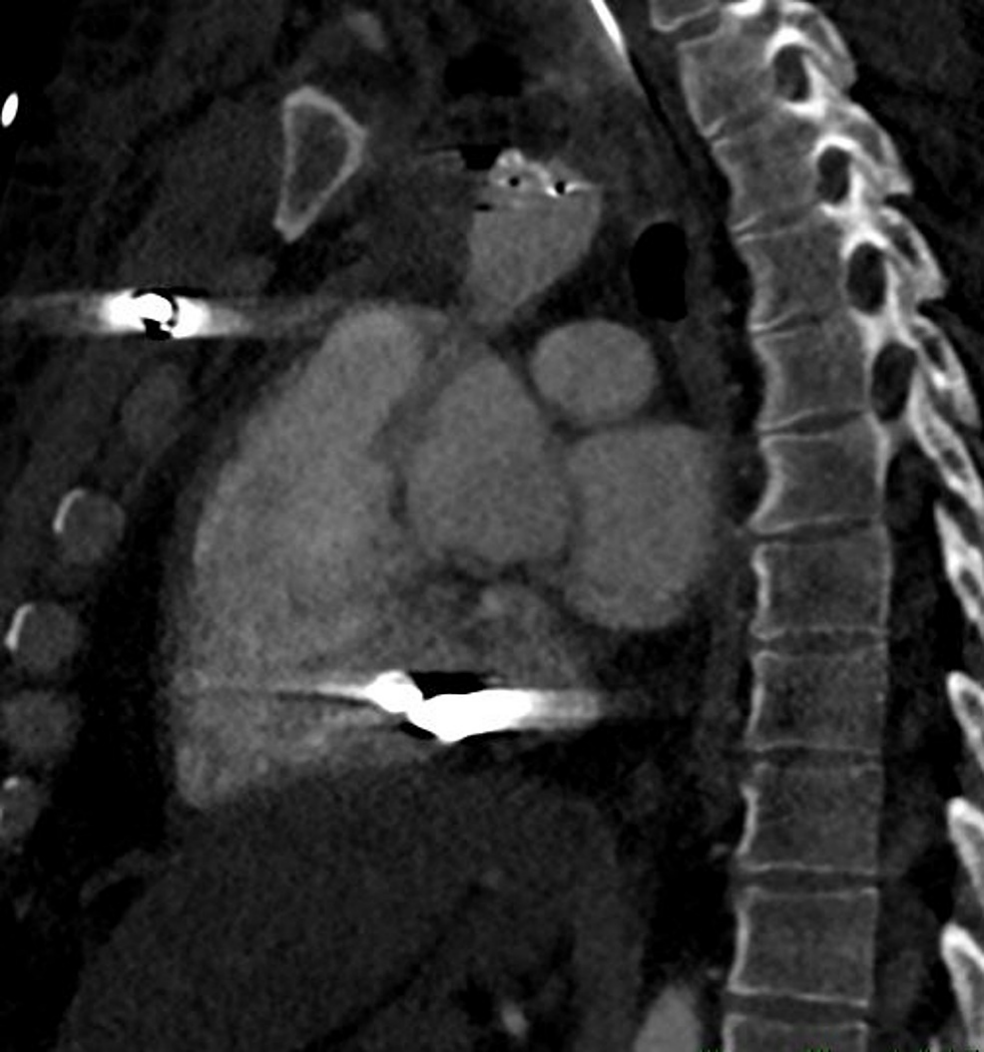
Lateral CT of the chest. Displays a hyperattenuated signal at the location of the bullet within the inferior aspect of the pericardium. Additionally, a second bullet is visualized by the hyperattenuated signal in the anterior thorax. CT: computed tomography

**Figure 2:**
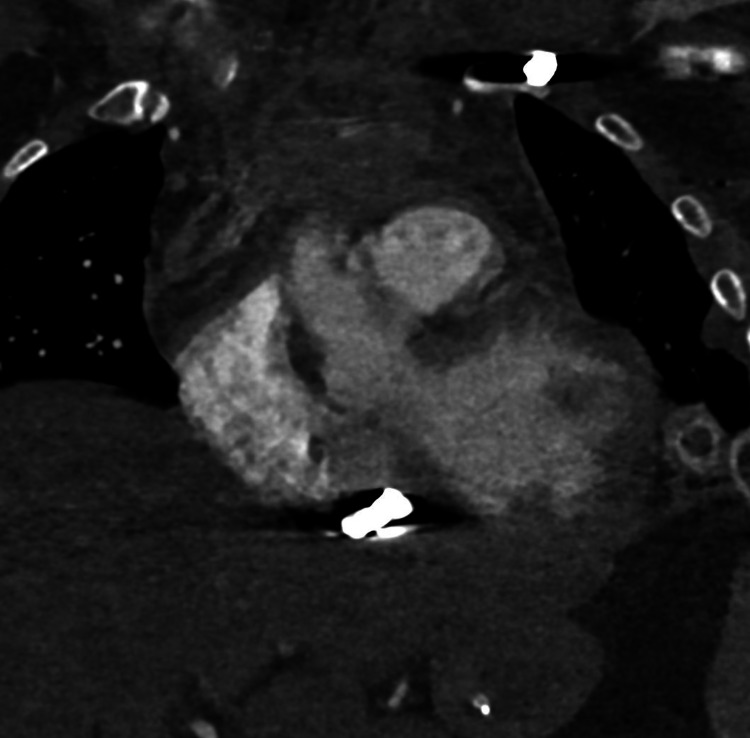
Anteroposterior CT of the chest. Displays two hyperattenuated signals: one localizing the bullet in the middle mediastinum inferior to the heart and the other located in the left shoulder, identifying the bullet that lacerated branches of the left subclavian vein. CT: computed tomography

**Figure 3:**
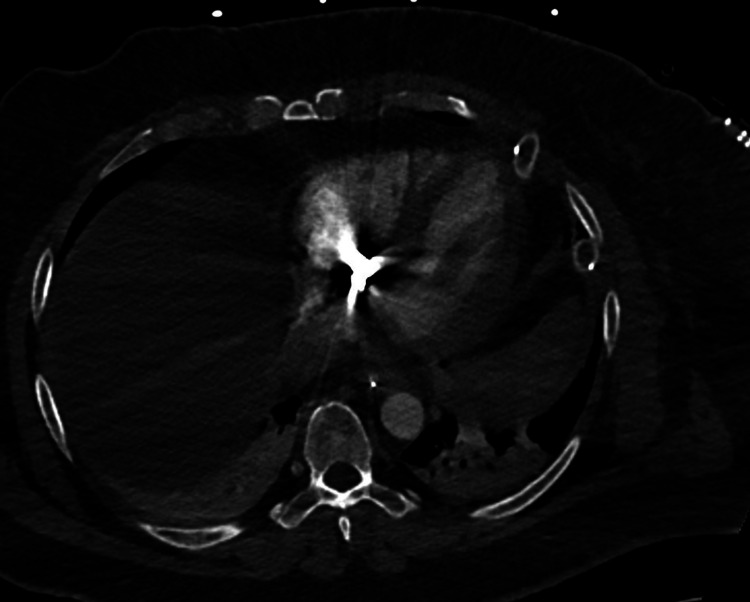
Transverse view of the chest. Displays a hyperattenuated signal in the pericardium of the right ventricle of the heart.

**Figure 4:**
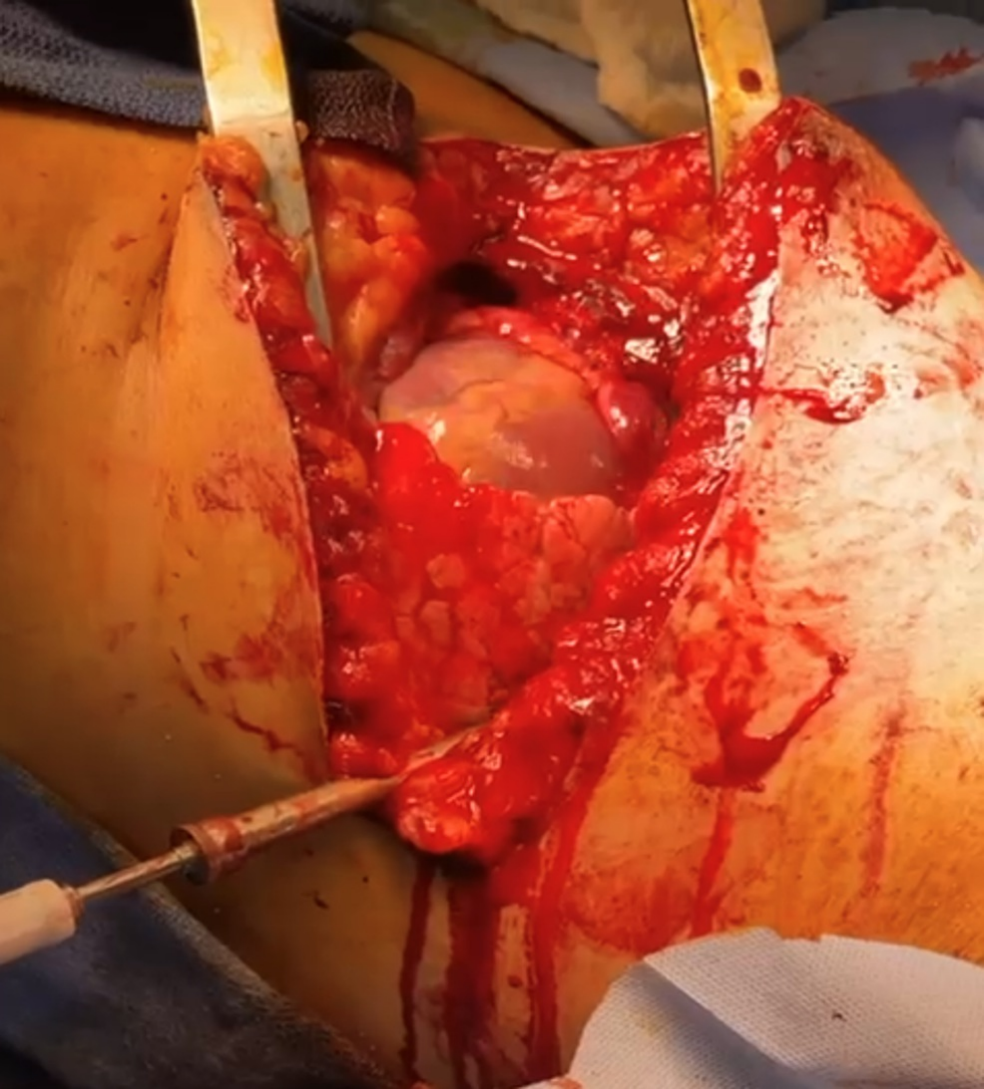
Transverse thoracotomy cut and the exposed heart.

**Figure 5:**
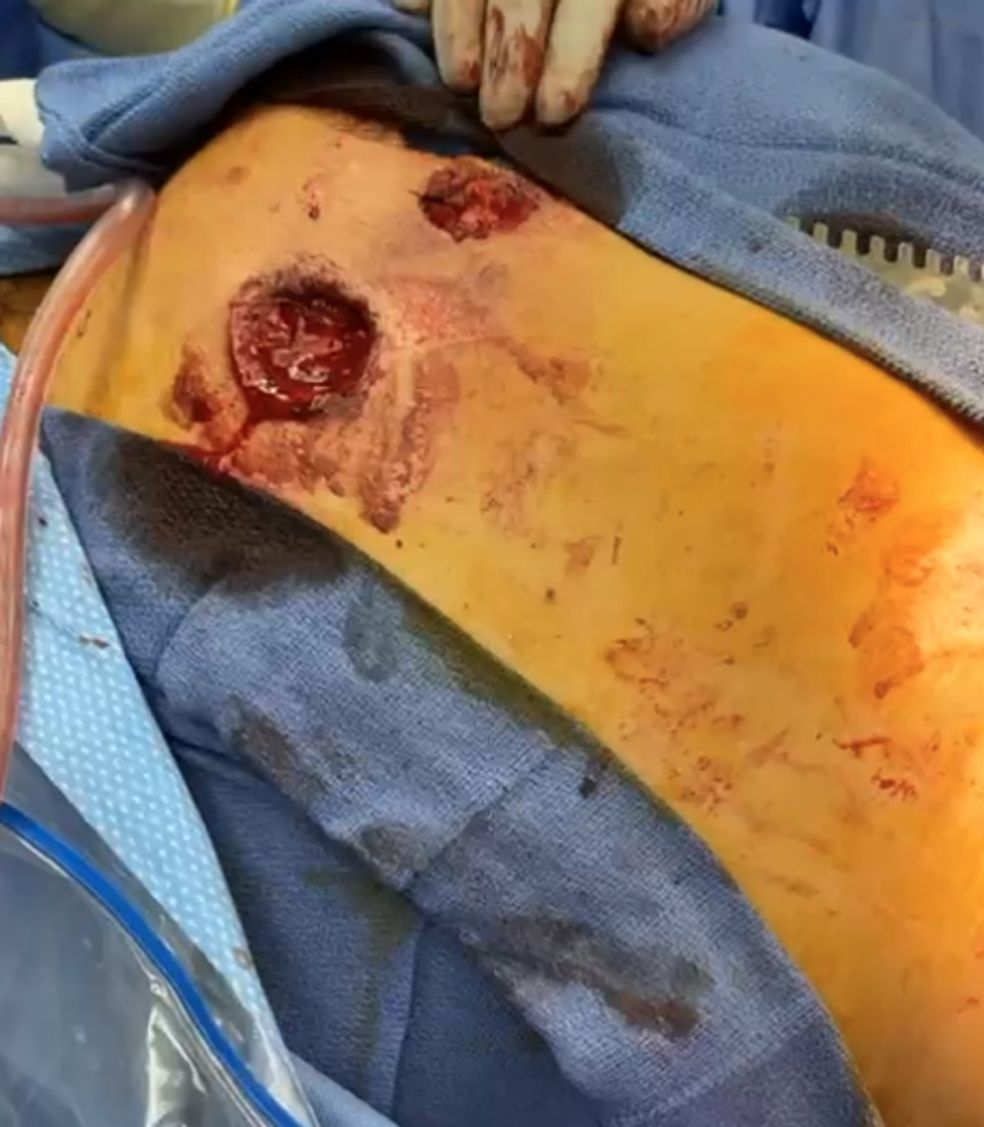
Abdominal gunshot wounds. Displays two high-caliber gunshot wounds to the anterior portion of the abdomen.

**Figure 6:**
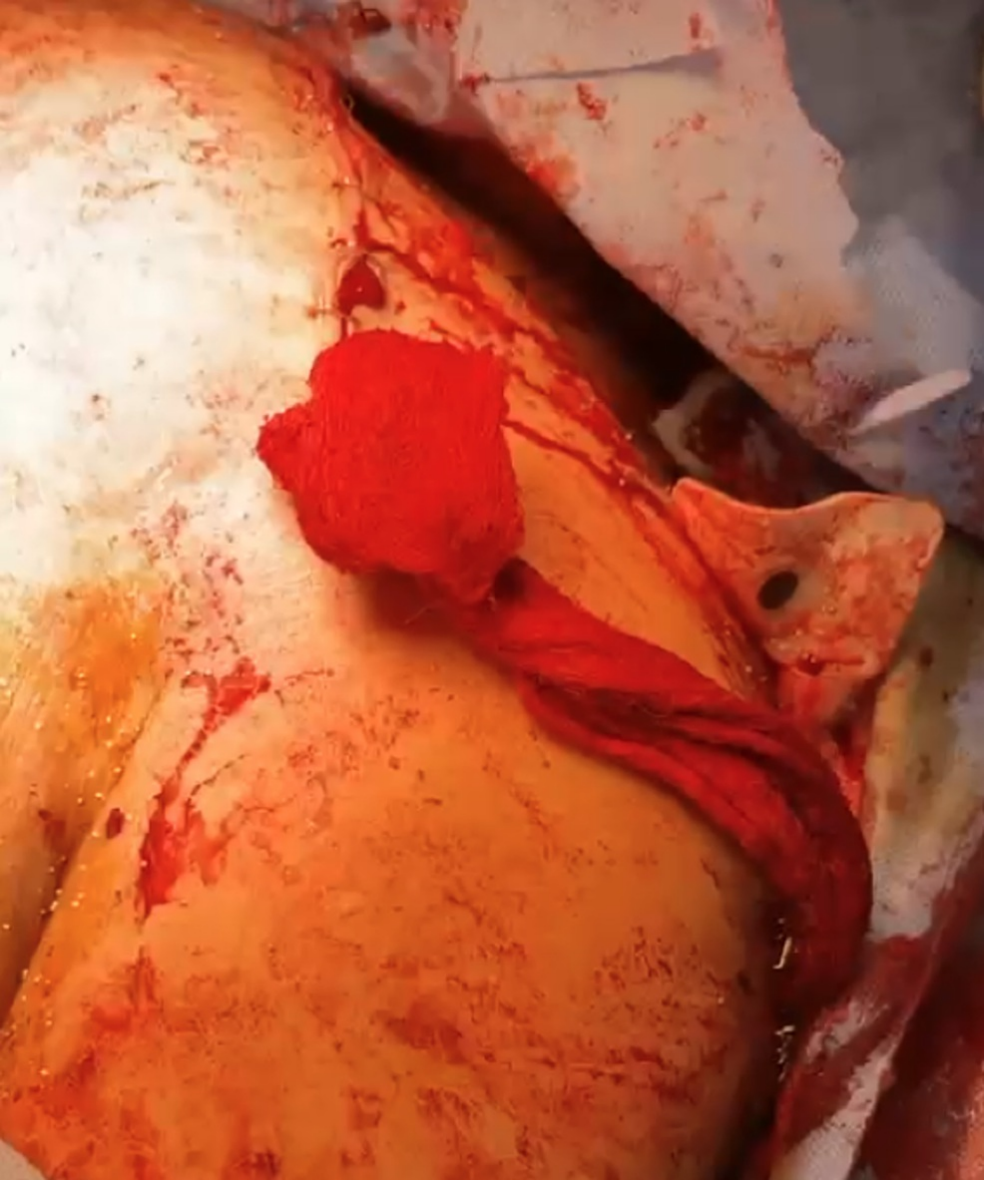
Left shoulder wound. Displays surgical closure after repair of the tributary of the left subclavian vein and removal of bone fragments from the shoulder injury.

Notably, throughout the DCR surgery, serial ABGs were monitoring the progress of the MTPs and trending the pH and coagulation values. In total, the patient was transfused with 18 units of pRBCs, 12 units of fresh frozen plasma, and three units of platelets with epinephrine and calcium for support of MTPs. CBC, coagulation, and ABG monitoring findings are listed in Table 1. During this operation, a continuous cardiac massage and warm saline were also administered to the heart. Additionally, an external convective warming blanket and additional cloth blankets were utilized, and the operating room temperature was raised to 80°F to prevent hypothermia and counteract the onset of this part of the lethal triad. The patient’s estimated blood loss was 5 L and 150 mL of urine was produced by the end of the surgery. The patient was also noted to be positive for coronavirus disease 2019. Radiological findings are demonstrated in Figures 1-3. Surgical visualizations are demonstrated in Figures 4-6.

**Table 1:** Complete blood count, coagulation, and arterial blood gas monitoring. *Arterial blood gas monitoring during damage control resuscitation surgery. WBC: white blood cell; RBC: red blood cell; Hgb: hemoglobin; INR: international normalized ratio; PT: prothrombin time; PTT: partial thromboplastin time

	09:20	09:42*	09:52*	10:12*	10:49*	11:32*	12:46	12:51	15:10
WBC (10^9^/L)	12.3	-	-	-	-	-	7.8	-	-
RBC (million/mm^3^)	4.53	-	-	-	-	-	5.20	-	-
Hgb	14.1	-	-	-	-	-	15.8	-	-
Platelets (10^3^/uL)	168	-	-	-	-	-	164	-	-
INR	1.6	-	-	-	-	-	1.2	-	-
PT (seconds)	19.2	-	-	-	-	-	15.2	-	-
PTT (seconds)	54	-	-	-	-	-	32	-	-
pH	-	<6.81	<6.81	7.02	7.30	7.46	-	7.37	7.41
pCO_2_ (mmHg)	-	69	54	40	35	38	-	41	38
pO_2_ (mmHg)	-	81	107	78	96	123	-	166	-
HCO_3 _(mEq/L)	-	-	-	10.3	17.2	27	-	23.7	24.1

## Discussion

This case describes a trauma patient exhibiting the lethal triad due to hemorrhage. Notably, the acidotic component was in an extreme range (pH <6.8). Lactic acidosis arises in the setting of massive hemorrhage as a consequence of the hypoperfusion of peripheral tissues due to blood loss. Ischemic tissue employs anaerobic metabolism to survive, producing lactic acid as a byproduct. Alterations to blood pH further complicate perfusion because of the effects of acid-base disturbances on oxygen delivery. The Bohr effect decreases the binding capacity of hemoglobin for oxygen, causing oxygen distribution to be suboptimal.

Hemostatic dysfunction is known to be directly correlated to the level of acidosis [8]. It is understood that enzymes and proteins operate optimally at specific, defined margins of temperature and pH. This hemostatic impairment, when coupled with hemorrhage in a trauma patient, complicates management.

Acidosis with a pH of <7.0 in the setting of trauma has been shown to increase the mortality rate threefold [9]. A lower threshold of acidosis below which there is certain mortality has not been found. The correction of this patient’s acidosis over the course of the OR procedure likely contributed to his survival.

Hypothermia (core body temperature of <35°C) in a hemorrhaging trauma patient arises due to massive blood extravasation that impairs the distribution of blood which typically aids thermoregulation. Furthermore, acidosis in a hemorrhaging patient impairs cardiac contractility, further decreasing the ability of blood to distribute throughout the body and provide warmth [10,11]. Hypothermia impairs the normal function of proteins involved in the coagulation cascade and can lead to mortality. Trauma patients with a core body temperature below 32.8°C have been observed to have a 100% mortality [12]. In our patient, the OR temperature was maintained above 80°F and a convective warming system was employed to prevent hypothermia. Postoperatively, the patient’s core temperature was measured at 37°C.

An international normalized ratio (INR) of >1.6 has been found to increase the odds of mortality [13]. This case had a borderline INR at presentation (1.6) that was corrected to 1.2 by the end of DCR and MTP. The timely correction of his coagulopathy was also important for the successful management of his critical condition.

In addition to the lethal triad, the patient required an open ED thoracotomy, which, in the setting of patients with multiple traumatic locations of injury such as in this case, has been found to have a survival rate of 0.7% [14]. This low survival rate in combination with the increased mortality from the presence of the lethal triad with severe acidosis emphasizes the complicated nature of this case.

## Conclusions

Trauma cases with severe acidosis complicating significant penetrating traumatic injuries in which the patient survives and has a steady, uncomplicated course of improvement are uncommon. This case reiterates the importance of MTP and management of the lethal triad in aiding patient survival. We demonstrate the use of MTP to correct the patient’s severe acidosis while managing core body temperature and coagulopathy.
